# Flexural Strengthening of Large-Scale RC Beams with Nonprestressed and Prestressed CFRP Using Different Anchorages

**DOI:** 10.3390/polym14245498

**Published:** 2022-12-15

**Authors:** Hai-Tao Wang, Zhi-Ning Bian, Guo-Wen Xu, Min-Sheng Chen, Hao Xiong, Sai-Sai Liu

**Affiliations:** 1 College of Civil and Transportation Engineering, Hohai University, Nanjing 210098, China; 2 China Construction Eighth Engineering Division Co., Ltd., Shanghai 200122, China; 3 Research Center of Shanghai Carbon Fiber Composite Application Technology in Civil Engineering, Shanghai 200122, China

**Keywords:** flexural strengthening, RC beam, anchorage method, CFRP type, prestressing

## Abstract

Externally bonded carbon-fiber-reinforced polymer (CFRP) technology can be used by different methods based on the anchorage device, CFRP type, and prestressing/nonprestressing. However, a direct comparison between the strengthening efficacies of different methods is still lacking. Seven large-scale RC beams were tested in this study to investigate the influences of the anchorage method, CFRP type, prestress, and prestressing system on the flexural strengthening efficacy of RC beams. The test results showed that the ultimate load increased by 38.3%, whereas the cracking and yielding loads were slightly affected when the anchorage method was enhanced from CFRP U-wraps to wedge-clamp anchors. The CFRP plate and CFRP sheet could provide a rather close flexural strengthening efficacy under the same CFRP strengthening amount. Compared to the nonprestressed CFRP plate, the prestressed CFRP plate was highly superior in improving the flexural behavior of RC beams. The cracking, yielding, and ultimate loads of the prestressed CFRP-strengthened specimens were 57.1%, 22.9%, and 5.9%, respectively, higher than those of the nonprestressed CFRP-strengthened specimen with an effective anchorage. The two types of prestressing systems based on the adhesive-friction anchor and wedge-clamp anchor were proven to be effective for flexural strengthening of RC beams with prestressed CFRP plates, and they could provide almost the same strengthening effect.

## 1. Introduction

In civil infrastructures, many reinforced concrete (RC) structures cannot meet structural requirements. Cracking could be caused by many combined factors, such as concrete carbonation, steel corrosion, excessive deflections due to prestress loss, shrinkage, and creep of concrete [1,2]. Strengthening these infrastructures has become necessary to increase structural safety and service life. In past decades, the strengthening technique based on fiber-reinforced polymer (FRP) has been an acceptable technique for strengthening RC structures due to advantages, such as the light weight, high strength, good corrosion, and fatigue resistance of FRP materials [3,4,5,6,7,8,9,10]. Among the different types of FRP materials, carbon–FRP (CFRP) is preferred, and the application forms of the CFRP in the strengthening of RC structures mainly include externally bonded (EB) CFRP laminates [11,12,13,14,15,16], near-surface-mounted (NSM) CFRP bars/strips [17], and externally prestressed CFRP bars/cables [18]. Among these strengthening methods, EB–CFRP technology is most widely used for strengthening RC structures. Extensive studies have been carried out on the flexural behavior of RC beams strengthened with EB–CFRP. When using the EB–CFRP technique for strengthening RC structures, one of the main issues is the premature debonding of the CFRP. When debonding occurs, the tensile strain of the CFRP is low, meaning that its tensile strength cannot be fully used [19,20]. In addition, many studies have shown that the ultimate capacity of strengthened RC beams can be improved by using CFRP laminates. In contrast, the flexural performance under normal service conditions slightly increases [21,22].

There are usually two types of methods used to postpone and even prevent the debonding process. The first method is to limit the debonding strain of the CFRP, which greatly decreases the allowable improvement of the flexural-load-carrying capacity [20]. The other method is to use an anchorage device at two ends of the CFRP laminate [23]. Different anchorage devices have been used in studies, such as mechanically fastened metallic anchors, U-jacket anchors, and FRP spike anchors [23,24,25,26,27]. Studies have shown that the use of FRP U-jackets at the FRP ends could change the failure mode from concrete cover separation and end-debonding to intermediate crack-induced (IC) debonding at higher loads, and the FRP U-jackets along the beam length at certain spacing could postpone the occurrence of IC debonding [24,28,29]. However, a study conducted by Khan et al. [30] found that the ultimate load-carrying capacity of the specimens strengthened with CFRP sheets increased by only 5.8% using CFRP wraps at two ends of the CFRP sheets, indicating that CFRP wraps had little effect on improving the strengthening efficacy. Spadea et al. [31] used a U-shaped steel anchor to improve the flexural behavior of RC beams strengthened with CFRP plates and found that the CFRP strain reached a 67% enhancement over the unanchored specimen. A study conducted by Wu and Huang [25] showed that specimens strengthened with 2-ply and 4-ply sheets failed due to CFRP rupture when combinations of EB–FRP and mechanical steel fasteners were used. Recently, Eslami et al. [32] developed a novel anchorage system of CFRP sheets by squeezing CFRP sheets into the groove at the ends of RC beams, and test results confirmed the efficacy of the proposed end anchorage technique. Generally, although several novel anchorage methods have been developed, the widely used anchorage methods in practical engineering are still FRP U-jackets and mechanically fastened steel plates. However, more studies should be conducted to assess the anchorage performance in large-scale girders with beam widths greater than 300 mm since most existing studies have used relatively narrow beam widths [23].

To increase the strength usage of the CFRP laminate and improve the structural behavior under normal service conditions, many studies have been carried out on the use of the prestressed CFRP laminate, and the effectiveness on flexural strengthening of RC beams has been validated [3,33,34,35,36,37,38]. The prestressing method is critical for the prestressed CFRP strengthening technique. Different methods have been developed to tension CFRP laminates. The first method is an indirect method. The strengthened beam is first cambered before bonding the CFRP, and then prestress is applied by releasing the camber after bonding and curing the CFRP [39]. However, the application of prestress is difficult, and the prestressing level is low in this method. These drawbacks limit its application in practical engineering [21,38,40]. The second method is to prestress the CFRP laminate using an independent external frame [33,34,41,42,43], which also belongs to an indirect method. After tensioning the CFRP, the CFRP is bonded on the surface of the member and then cured, followed by cutting the CFRP from the external anchors. This method is widely used in small-scale beams in the laboratory, but using heavy external equipment could be unrealistic in practical engineering [40]. The third method is to directly tension the CFRP laminate against the strengthened beam itself [3,21,38]. This method can be conveniently used in practical engineering [21]. However, the majority of previous studies have used the second method. Therefore, more studies need to be conducted on developing practical prestressing systems and evaluating their efficacy in strengthening RC beams.

Although many studies have investigated the flexural behavior of RC beams strengthened with nonprestressed and prestressed CFRP materials, a literature review found that further studies should be conducted to promote the application of the EB–CFRP technique. First, small-scale specimens were always used in previous studies to verify the anchorage efficiency of anchorage methods (especially FRP U-jackets and mechanically fastened steel plates). The anchorage efficiency should be further investigated using large-scale specimens. Second, most prestressing methods used in previous studies required a special reaction frame, which could be unrealistic in practical applications. More prestressing systems for practical applications should be presented and validated. Third, when RC beams are strengthened with the EB–CFRP technique, some choices have to be made, such as a CFRP plate or sheet, the CFRP to be prestressed or not, and the anchorage method/device of the CFRP. However, a direct comparison between the strengthening efficacies of different EB–CFRP methods is still lacking in the references. Therefore, an experimental study was conducted using seven large-scale RC specimens in this study. Three different anchorage methods were compared, and two practical prestressing systems with different anchors and tensioning methods were used. The effects of the anchorage method, CFRP type, prestress, and prestressing system on the flexural strengthening efficacy of RC beams were compared and analyzed. The findings of this paper can provide a reference for the engineering application of the EB–CFRP technique.

## 2. Experimental Program

### 2.1. Materials Properties

The concrete used in this study was commercial concrete with a grade of C30. Five cubic coupons with a length of 150 mm were cured for 28 days under the same outdoor conditions as the beam specimens. The average compressive strength obtained by the coupon tests [44] was 26.8 MPa, which was lower than 30 MPa due to the poor curing environment. All the steel rebars were of HRB400 grade. Table 1 lists the main mechanical properties obtained by the coupon tests [45] of the longitudinal tensile rebars. Two types of CFRP materials were used to strengthen RC beams, i.e., a CFRP plate and a CFRP sheet. The nominal width and thickness of the CFRP plate were 100 mm and 1.4 mm, respectively. The nominal width and thickness of one layer of the CFRP sheet were 200 mm and 0.167 mm, respectively. The main mechanical properties of the CFRP plate and sheet tested according to the relevant standard [46] are listed in Table 1. Two epoxy adhesives were used to bond the CFRP plate and CFRP sheet on the concrete surface. The mixture ratio of parts A and B was 2:1 by weight for the two types of adhesives. The main mechanical properties of the epoxy adhesives provided by the manufacturer are shown in Table 1.

### 2.2. Specimen Design

**Figure 1:**
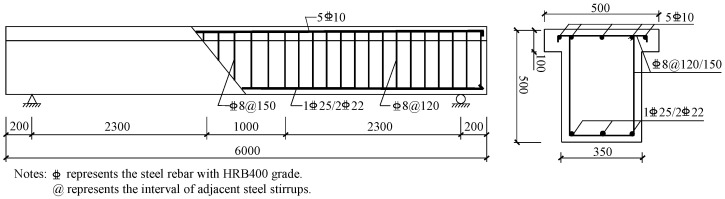
Schematic of an RC beam (unit in mm).

Large-scale RC beams with a T-shaped section were used as specimens in this study. The schematic of an RC beam is shown in Figure 1. The total length of the specimen was 6000 mm, and its total height was 500 mm. The widths of the web and the flange were 350 mm and 500 mm, respectively. The height of the flange was 100 mm. The concrete cover was 20 mm. Two rebars with a diameter of 22 mm and one rebar with a diameter of 25 mm were arranged in the tension zone, and five rebars with a diameter of 10 mm were arranged in the compression flange. The reinforcement ratio was 0.76%, which was much lower than the balanced ratio of 2.05%. Based on the code [47], the diameter of the steel stirrups was 8 mm, and they were arranged in the pure-bending and the flexure-shear segments at intervals of 150 mm and 120 mm to provide sufficient shear capacity in all specimens, as shown in Figure 1.

**Figure 2:**
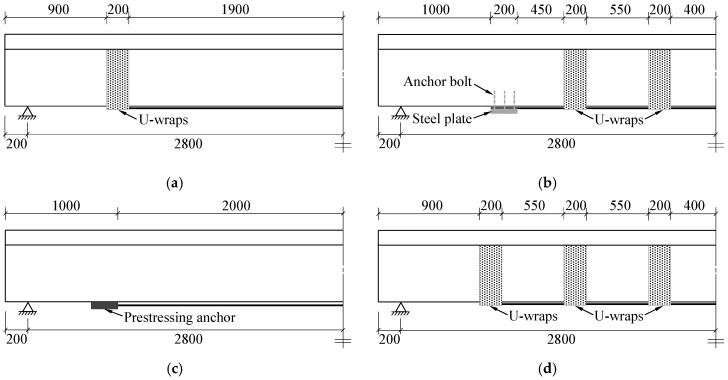
Anchorage methods of the specimens (unit in mm): (**a**) BS-1; (**b**) BS-2; (**c**) BS-3, BP-1, and BP-2; (**d**) BS-4 (unit in mm).

A total of seven specimens were designed and tested in this study, including one unstrengthened specimen and six strengthened specimens with nonprestressed and prestressed CFRP materials. All strengthened specimens were designed based on the condition of the same CFRP strengthening amount, i.e., the values of *A_f_* and *E_f_* were the same (*A_f_* and *E_f_* are the cross-sectional area and Young’s modulus of the CFRP, respectively). Four variables were considered in this study, including the anchorage method of the nonprestressed CFRP plate, the CFRP type, prestress, and the prestressing system of the prestressed CFRP plate. The strengthening details of the specimens are listed in Table 2. In the table, specimen BU was the unstrengthened beam. Specimens BS-1, BS-2, and BS-3 stood for the beams strengthened with a nonprestressed CFRP plate using different anchorage methods, as shown in Figure 2a–c. The CFRP plate in specimen BS-1 was anchored only using CFRP U-wraps at two ends, while that in specimen BS-2 was anchored using both steel plates at two ends and four CFRP U-wraps at the intermediate part of the specimen. Two steel plates with a length of 200 mm, a width of 200 mm, and a thickness of 12 mm were fixed using six chemical anchor bolts with a diameter of 16 mm. The CFRP plate in specimen BS-3 was anchored using wedge-clamp anchors, which were the same as those used in specimen BP-2. Specimen BS-4 denoted the beam strengthened with three layers of CFRP sheets, which were anchored using six CFRP U-wraps at the two ends and the intermediate part, respectively, as shown in Figure 2d. Each CFRP U-wrap used in specimens BS-1, BS-2, and BS-4 included three layers of CFRP sheets. Specimens BP-1 and BP-2 were the beams strengthened with a prestressed CFRP plate, but the prestressing systems used in the two specimens were different. Adhesive-friction anchors and wedge-clamp anchors were used in specimens BP-1 and BP-2, respectively. The designed initial tensioning strain of the CFRP plate in both specimens was 4500 με, i.e., approximately 738 MPa prestress was applied in the CFRP plate.

### 2.3. Prestressing Anchor and Tensioning Method

**Figure 3:**
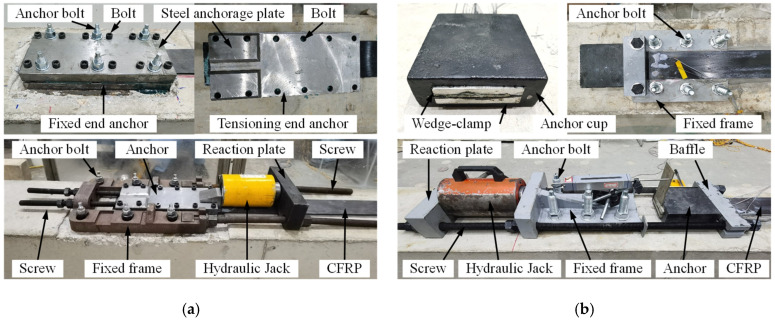
Prestressing systems used in the tests: (**a**) adhesive-friction anchor and (**b**) wedge-clamp anchor.

The prestressing system is crucial for the prestressed CFRP strengthening technology. This study used two types of prestressing systems to verify and compare their strengthening efficacy. The adhesive-friction anchors, consisting of two thick steel anchorage plates with grooves on the inner surface, were used in specimen BP-1. The CFRP plate and the adhesive were pressed by two steel anchorage plates using twelve high-strength bolts to implement the anchorage of the CFRP plate, as shown in Figure 3a. The anchor at the fixed end was directly installed on the beam surface using six chemical anchor bolts. The anchor at the tensioning end was connected to a steel fixed frame using two high-strength screws. The wedge-clamp anchors, including one anchor cup and two wedge-clamps in each anchor, were applied in specimen BP-2, as shown in Figure 3b. A steel fixed frame held the anchor at the fixed end, and the anchor at the tensioning end was connected to a steel fixed frame using two high-strength screws and a steel baffle. The steel fixed frames were installed on the beam surface using six chemical anchor bolts.

Apart from anchors, tensioning methods used in the two specimens were also different. As shown in Figure 3, the adhesive-friction anchor was pushed by a hydraulic jack, whereas the wedge-clamp anchor was pulled by a hydraulic jack. They were called the reverse tensioning method and the positive tensioning method, respectively, in this paper. Compared to the positive tensioning method, the advantage of the reverse tensioning method was that the tensioning process did not take up the space at the beam end, resulting in greater applicability in the operation space. Therefore, the reverse tensioning method is especially suitable for application in a narrow space.

**Figure 4:**
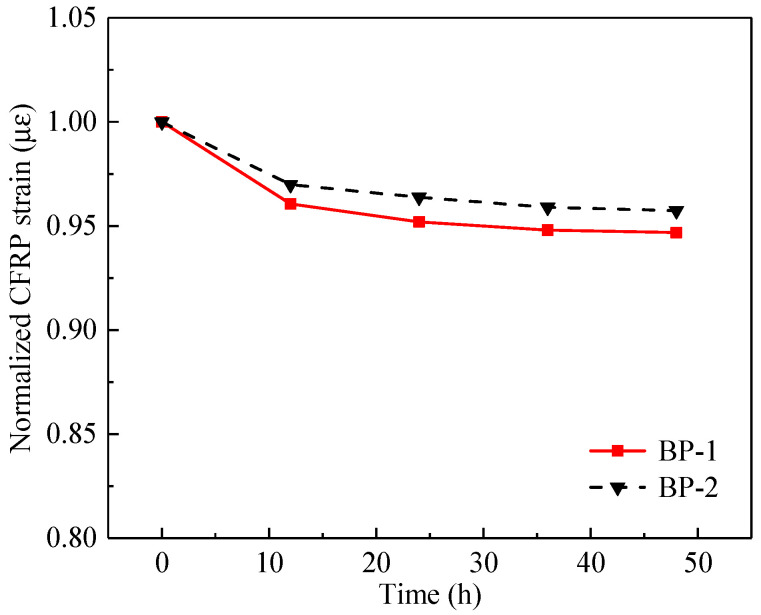
Variations of the normalized CFRP strain with time.

During tensioning of the CFRP plate, a pre-tensioning procedure was first conducted to check the prestressing system. Next was the unloading of the pre-tensioning stress and the adhesive application. In the formal tensioning stage, the final tensioning strain was applied at 1.05*ε*_con_ (*ε*_con_ was the designed tensioning strain of the CFRP plate). An over-tensioning of 0.05*ε*_con_ aimed to compensate for the potential prestress loss according to the relevant standard [48]. After the prestressing procedure, the hydraulic jack and the extruded adhesive were removed. Figure 4 shows the variations in the normalized CFRP strain with time, where the normalized CFRP strain is defined as the ratio between the time-dependent strain of the CFRP plate after removing the jack and the initial tensioning strain. It can be seen that the reduction rate of the CFRP strain decreased gradually with time, and the reductions in the normalized CFRP strain of the two specimens were 5.3% and 4.3%, respectively, after 48 h.

### 2.4. Test Procedures and Instruments

**Figure 5:**
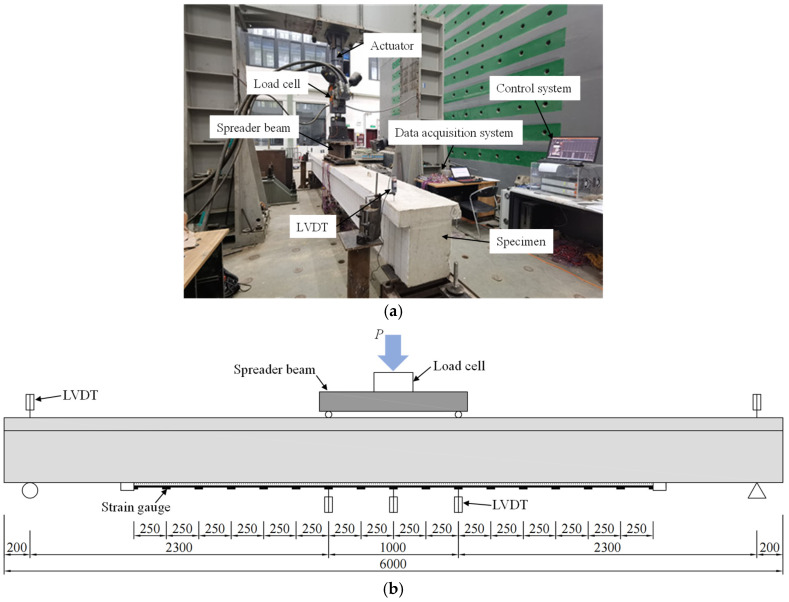
Test setup of the specimen: (**a**) loading photo and (**b**) schematic of loading and instruments (unit in mm).

After all specimens were cured for 1 week, tests were conducted in a hydraulic-servo testing system (walter + bai, Schaffhausen, Switzerland) with a maximum capacity of 500 kN. The specimens were simply supported and loaded under four-point bending with a distance of 1000 mm between the applied loads. The clear span of each specimen was 5600 mm. The length of each bending-shear segment was 2300 mm, and the ratio between the shear span and effective depth [49] was 5. The test setup is shown in Figure 5. The loading procedure included the preloading stage and the loading stage. In the preloading stage, all specimens were first loaded to 2 kN to check the testing systems and instruments. In the loading stage, the specimens were loaded at a stroke rate of 0.9 mm/min before yielding of the tension steel rebar, and then the loading rate was increased to 3.0 mm/min after yielding of the tension steel rebar. The loading procedure was not stopped until the concrete at the compressive zone was finally crushed for all specimens. However, the specimens were judged to reach the ultimate state once one of the following conditions was first met: (1) the concrete in the compression zone was crushed; (2) the end-debonding of the CFRP plate, i.e., anchorage failure, occurred; and (3) the CFRP plate completely fractured.

Linear variable displacement transducers (LVDTs) and strain gauges were used to measure the displacements and strains of each specimen, as shown in Figure 5b. A total of five LVDTs were mounted to measure the displacements at the mid-span, two loading points, and two supporters. Seventeen strain gauges were bonded on the surface of the CFRP plate at an interval of 250 mm. The displacements and strains were collected using an automatic data acquisition system, while the loads were recorded directly using the testing system.

## 3. Results and Discussion

### 3.1. Experimental Observation and Failure Mode

**Figure 6:**
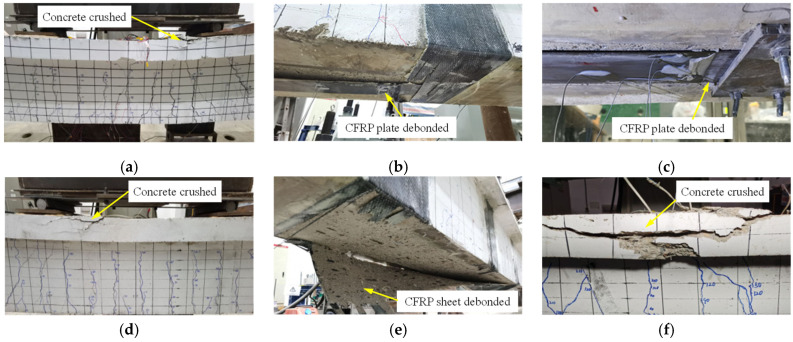
Typical failure photos: (**a**) BU, (**b**) BS-1, (**c**) BS-2, (**d**) BS-3, (**e**) BS-4, and (**f**) BP-2.

Specimen BU showed a typical flexural failure of the RC beam (Figure 6a). Four specimens strengthened with nonprestressed CFRP plates or sheets had two different failure modes: end-debonding and concrete crushing. For specimens BS-1, BS-2, and BS-4, which were anchored by U-wraps or steel plates, progressive intermediate debonding occurred after the tensile steel rebar yielded. Finally, the specimens suddenly failed due to the end-debonding at one of the ends of the CFRP, as shown in Figure 6b,c,e. The end-debonding caused a great drop in the applied load, and the CFRP did not provide the strengthening effect anymore. However, the end-debonding of the CFRP plate was avoided in specimen BS-3, which was anchored by the wedge-clamp anchors. Thus, specimen BS-3 did not fail until the compressive concrete was crushed, as shown in Figure 6d. In conclusion, the anchorage method has a significant effect on the failure mode of CFRP-strengthened RC beams.

Two specimens strengthened with prestressed CFRP plates exhibited similar experimental phenomena and failure modes. Following the yielding of tensile steel rebars, intermediate debonding of the CFRP plate occurred soon and gradually propagated toward the two ends for both specimens. Similar to specimen BS-3, the CFRP plates in specimens BP-1 and BP-2 were still supported by anchors, even though they had debonded completely between two anchors. The CFRP plate could continue to bear the increasing load until the compressive concrete was crushed (Figure 6f). This verified the two types of anchors could provide a reliable anchorage effect.

### 3.2. Flexural Strengthening Effect

**Figure 7:**
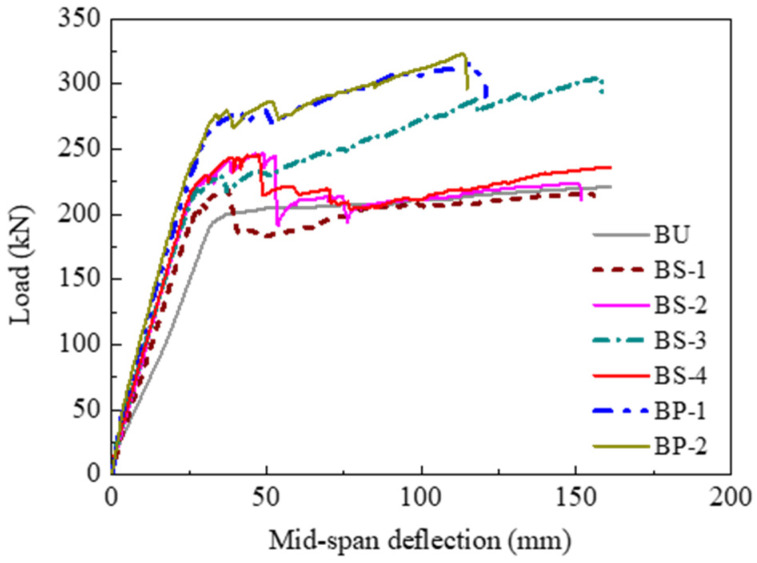
Comparisons of load–deflection curves.

The load versus mid-span deflection curves of all specimens are plotted in Figure 7. The CFRP used in all strengthened specimens had the same strengthening amount, allowing the direct comparison of the flexural strengthening efficacy of different methods based on the EB–CFRP technique. Generally, all the load–deflection curves showed three different stages: the uncracking stage, the post-cracking stage, and the post-yielding stage. At the uncracking stage, both nonprestressed strengthening and prestressed CFRP strengthening led to a slight improvement in the flexural stiffness compared to the unstrengthened specimen. This result was primarily caused by the stiffness of the CFRP being rather low compared to the large-scale beam section. After the tensile concrete cracked, the improvement in the flexural stiffness became remarkable using CFRP strengthening, and prestressed CFRP strengthening could provide a higher flexural stiffness than nonprestressed CFRP strengthening. After the tensile steel rebars yielded, the load–deflection curves of the strengthened specimens presented an obvious post-yielding stiffness due to the linear-elastic behavior of the CFRP, which allowed the CFRP to carry an increasing load even if the steel rebars had yielded. However, the performance of the post-yielding stage for the strengthened specimens was affected significantly by the anchorage method and the prestress of the CFRP plate. The specimens with concrete crushing failure exhibited a much longer post-yielding stage than those with end-debonding failure. Under the same effective anchorage method, the prestressed CFRP-strengthened specimens BP-1 and BP-2 exhibited a shorter post-yielding stage compared to the nonprestressed CFRP-strengthened specimen BS-3.

The main characteristic loads of all specimens are listed in Table 3. In the table, the characteristic loads *P_cr_*, *P_y_*, and *P_u_* represent the cracking, yielding, and ultimate loads, respectively. The parameters *α_cr_*, *α_y_*, and *α_u_* represent the ratios between the cracking, yielding, and ultimate loads of the strengthened specimens and those of the unstrengthened specimen, respectively. Generally, the use of both the CFRP plate and the CFRP sheet could enhance the flexural behavior of RC beams. However, the strengthening efficacies were significantly different, although the CFRP strengthening amount was the same in all strengthened specimens. The effects of different parameters on the strengthening efficacy were analyzed and are discussed later.

#### 3.2.1. Influence of the Anchorage Method

**Figure 8:**
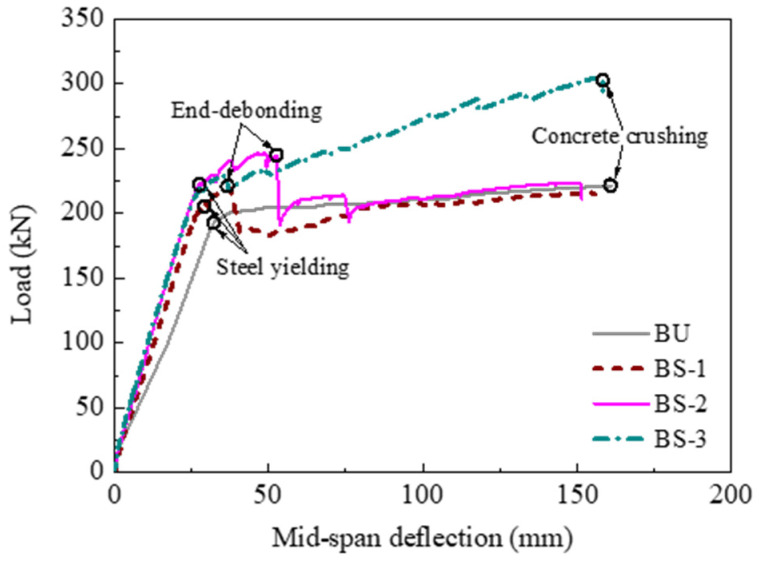
Influence of the anchorage method.

The influence of the anchorage method on the strengthening effect was investigated by comparing the test results of specimens BS-1, BS-2, and BS-3. Figure 8 presents the comparisons of the load–deflection curves. The flexural behavior of the three specimens was enhanced gradually with stronger anchorage devices. The yielding and ultimate loads of specimen BS-2 were 15.7% and 11.6% higher than those of specimen BU, respectively. In comparison, the yielding load of specimen BS-1 was only 7.9% higher, and the ultimate load did not increase compared to specimen BU due to premature end-debonding. When wedge-clamp anchors were used in specimen BS-3, the flexural strengthening efficacy significantly improved. The yielding and ultimate loads of specimen BS-3 increased by 13.6% and 37.8%, respectively, compared with those of specimen BU. Specimen BS-3 could carry a 23.5% (i.e., approximately 58 kN) higher ultimate load than specimen BS-2. These comparisons demonstrated that the anchorage method affects the strengthening efficacy. Both the steel anchorage plates and the CFRP U-wraps were insufficient to provide an effective anchorage under the ultimate limit state, resulting in premature end-debonding of the CFRP plate. This finding was different from that in [24] with small-scale specimens. In contrast, the wedge-clamp anchors could avoid end-debonding, which was proven to be the most effective among the three anchorage methods. Therefore, an effective anchorage method should be used in engineering applications.

#### 3.2.2. Influence of the CFRP Type

**Figure 9:**
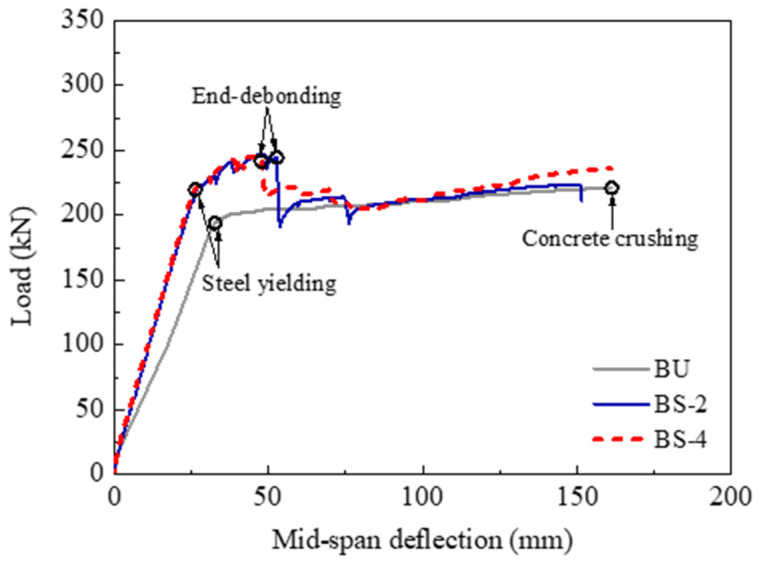
Influence of the CFRP type.

The experimental results of specimens BS-2 and BS-4 were used to investigate the influence of the CFRP type on the flexural strengthening effect. The two specimens had similar anchorage methods and almost the same CFRP strengthening amount. Figure 9 shows that the load–deflection curves of the two specimens approximately overlapped before the tensile steel rebars yielded. After yielding, the differences between the two curves were mainly attributed to the fluctuations caused by the CFRP debonding. The cracking, yielding, and ultimate loads were close for the two specimens, i.e., 26 kN versus 24 kN, 222.6 kN versus 221.6 kN, and 246.9 kN versus 246.7 kN, respectively. Therefore, the CFRP plate and CFRP sheet had almost the same strengthening efficacy in improving the flexural behavior of RC beams when the strengthening amount and the anchorage method were close.

#### 3.2.3. Influence of the Prestress

**Figure 10:**
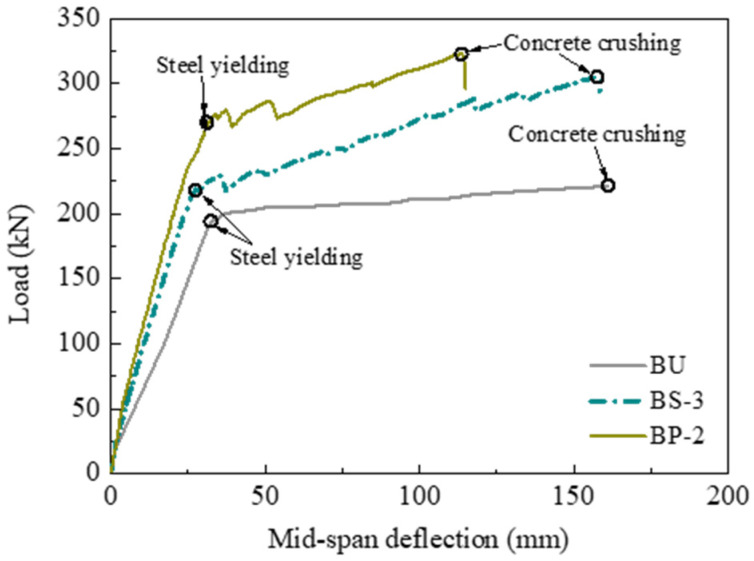
Influence of the prestress.

The influence of the prestress in the CFRP plate on the flexural strengthening efficacy was investigated using specimens BS-3 and BP-2. Figure 10 compares the load–deflection curves of the specimens. The cracking load of the RC beam significantly increased when an initial prestress of approximately 738 MPa was applied to the CFRP plate. The cracking load of specimen BP-2 was 2.75 times that of specimen BU, whereas that of specimen BS-3 was only 1.75 times that of specimen BU. Similarly, the yielding and ultimate loads of specimen BP-2 increased by 39.6% and 46.0%, respectively, while those of specimen BS-3 increased only by 13.6% and 37.8%, respectively, compared to specimen BU. The characteristic loads of specimen BP-2 could be further increased by 57.1%, 22.9%, and 5.9% compared with those of specimen BS-3. In conclusion, the prestressed CFRP plate has an obvious superiority in improving the flexural behavior of RC beams under both the serviceability limit state and the ultimate limit state compared to the nonprestressed CFRP plate.

#### 3.2.4. Influence of the Prestressing System

**Figure 11:**
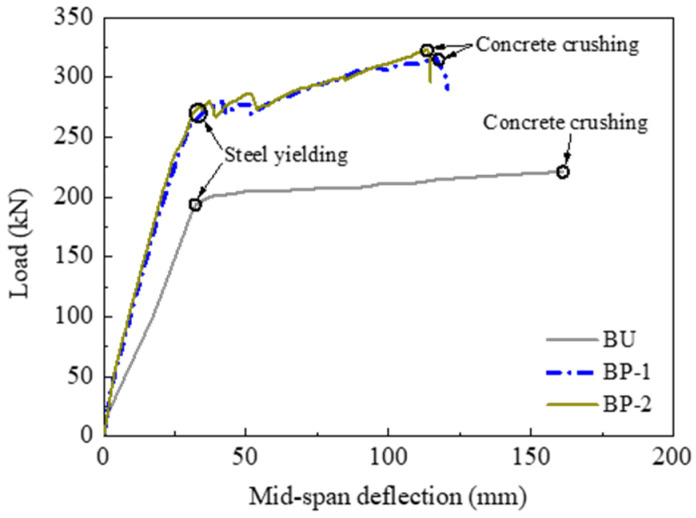
Influence of the prestressing system.

Specimens BP-1 and BP-2 were strengthened with prestressed CFRP plates using two types of prestressing systems. The comparisons of the load–deflection curves are shown in Figure 11. The flexural strengthening efficacies of the two prestressing systems were nearly the same in terms of the almost coincidental load–deflection curves and the rather close characteristic loads. For example, the yielding loads were 265.2 kN and 269.9 kN, while the ultimate loads were 320.9 kN and 323.1 kN, respectively, for the two specimens. The key to the prestressed CFRP strengthening method is the effective anchorage of the CFRP plate. This test found that the anchorage efficacy of both prestressing systems is reliable during the whole loading stage. Therefore, the CFRP plate could strengthen the RC beams until the concrete was crushed for both specimens. This analysis verified that both types of prestressing systems can be used in the flexural strengthening of RC beams with prestressed CFRP plates.

### 3.3. Load–CFRP Strain Curves

**Figure 12:**
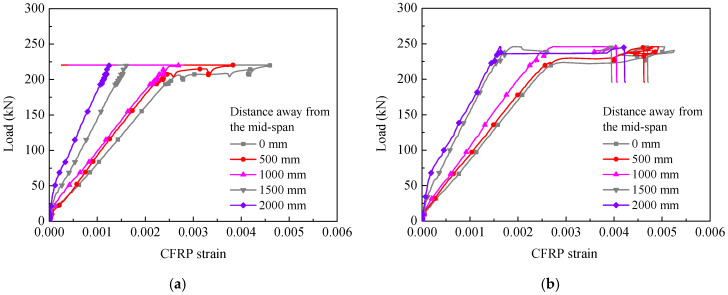

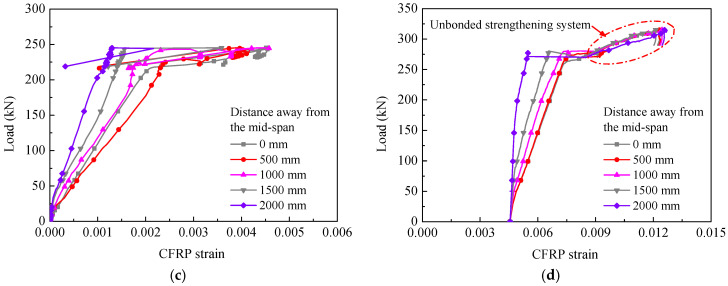
Typical load–CFRP strain curves: (**a**) BS-1, (**b**) BS-2, (**c**) BS-4, and (**d**) BP-2.

Figure 12 shows the load–CFRP strain curves of typical specimens. Similar to the load–deflection curves, the CFRP strain development with the applied loading also included three different stages. Before the cracking of the tensile concrete, the increase rate of the CFRP strain was slow with the increase in the applied load because the concrete, steel rebars, and the CFRP could carry the tensile force together at this stage. The strain development of the CFRP entered the second stage when the tensile concrete cracked. The increase rate of the CFRP strain became faster than that in the first stage, and an obvious turning point was observed in each curve. After the tensile steel rebars yielded, the CFRP strain increased quickly because the increased tensile force was resisted only by the CFRP. In this stage, the CFRP strains fluctuated along with the partial intermediate-debonding of the CFRP. For the specimens with end-debonding failure, the strains decreased sharply due to the end-debonding at failure. However, for the strengthened specimens with concrete crushing failure, after the bonded strengthening system was changed to the unbonded system, the strain development curves at different strain gauges almost overlapped until the compressive concrete was crushed, as shown in Figure 12d.

### 3.4. Ductility

The commonly used ductility index is represented by the ratio between the deflections at the ultimate load and the yielding load. Table 4 lists the ductility index of all specimens. In the table, Δ*_y_* denotes the mid-span deflection at the yielding load and Δ*_u_* denotes the mid-span deflection at the ultimate load. Generally, the anchorage method and prestress of the CFRP significantly affected the ductility index of CFRP-strengthened specimens. The values of the ductility index for specimens BS-1, BS-2, and BS-4 decreased by 65.5%–75.5% relative to specimen BU due to premature end-debonding failure. However, the ductility index of specimen BS-3 reached 5.73, which was close to that of specimen BU and much higher than that of the other strengthened specimens. This indicated that the CFRP-strengthened specimens can exhibit a rather good deformation capacity if an effective anchorage method is used to avoid end-debonding failure. In addition, the ductility of specimen BS-2 was higher than that of specimen BS-1, demonstrating that the combination of steel anchorage plates and CFRP U-wraps is more beneficial to ductility than CFRP U-wraps alone at the two ends. From specimens BS-3, BP-1, and BP-2, it can be seen that the ductility index reduced when the CFRP plate was prestressed. For example, the ductility index reduced from 5.73 to 3.64, i.e., a 36.5% reduction, when a 738 MPa prestress was applied to the CFRP plate.

### 3.5. CFRP Use

The CFRP use was evaluated by the ratio between the CFRP strain in the loading and its ultimate strain. Table 5 lists the CFRP strains at different loading stages and the corresponding use of all strengthened specimens. In the table, the CFRP strain was measured by the strain gauge bonded at the mid-span; *ε_f,y_*, *ε_f,_*_max_, and *ε_f,u_* represent the CFRP strain at the yielding load, the CFRP strain at failure, and the CFRP ultimate strain, respectively. Consequently, *ε_f,y_*/*ε_f,u_* denotes the CFRP use at the yielding load, while *ε_f,_*_max_/*ε_f,u_* denotes the CFRP use at failure. As shown in Table 5, for specimens BS-1, BS-2, and BS-4, the CFRP use was low at both yielding and failure stages, meaning that the high-strength superiority of the CFRP was not fully exploited in the entire loading stage. For example, for specimen BS-1, the CFRP use was exploited to only 15.9% at the yielding load and 27.2% at failure. When an effective anchorage was used in specimen BS-3, CFRP use at the yielding stage (i.e., 17.4%) did not improve but the use at the failure stage (i.e., 59.0%) remarkably increased. This analysis demonstrated that CFRP use is low, especially under the serviceability limit state, if the nonprestressed CFRP is used.

The specimens strengthened with the prestressed CFRP plates greatly increased the CFRP use in both yielding and failure states. Approximately half the strength of the CFRP plate was used at the yielding load, while the CFRP use could reach more than 70% at the failure state for specimens BP-1 and BP-2. Compared to the four nonprestressed CFRP-strengthened specimens, the CFRP use of specimens BP-1 and BP-2 at the yielding load increased by more than 160%. Moreover, the CFRP use at the failure state increased by approximately 23.7% compared with that of specimen BS-3. Therefore, applying prestress to the CFRP plate is an effective method for increasing the strength use of the CFRP under both the serviceability limit state and the ultimate limit state.

## 4. Theoretical Calculation

For the nonprestressed and the prestressed CFRP strengthening technique, a design code [50] has been proposed to guide practical applications. The ultimate capacity can be calculated according to the basic equations in the code [50]. In this study, the compressive zone of the concrete was located in the top flange due to a small reinforcement ratio. Therefore, the ultimate bending moment of the strengthened specimens can be calculated based on Equations (1) and (2):

(1) $$M_{u}=f_{c}b_{f}^{\prime }x\left(h_{0}-\frac{x}{2}\right)+f_{y}^{\prime }A_{s}^{\prime }\left(h_{0}-a^{\prime }\right)+\sigma_{f}A_{f}\left(h_{\mathrm{fe}}-h_{0}\right)$$

(2) $$f_{c}b_{f}^{\prime }x=f_{y}A_{s}-f_{y}^{\prime }A_{s}^{\prime }+\sigma_{f}A_{f}$$

where *M_u_* is the ultimate bending moment; *f_c_* is the concrete compressive strength; $b_{f}^{\prime }$ is the width of the flange; *x* is the height of the compressive concrete; *h*_0_ is the distance between the action point of the resultant force of tensile rebars and the compressive edge; $A_{s}^{\prime }$ and $f_{y}^{\prime }$ are the cross-sectional area and yielding stress of compressive rebars, respectively; *a*′ is the distance between the action point of the resultant force of compressive rebars and the compressive edge; *σ_f_* is the tensile stress of the CFRP laminate when the ultimate limit state is reached; *A_f_* is the cross-sectional area of the CFRP laminate; *h*_fe_ is the distance between the action point of the resultant force of the CFRP laminate and the compressive edge; and *A_s_* and *f_y_* are the cross-sectional area and yielding stress of tensile rebars, respectively. For the nonprestressed CFRP laminate, its tensile stress *σ_f_* can be determined using Equations (3)–(5):

(3) $$\sigma_{f}\;=\min\left\{f_{f},E_{f}\varepsilon_{\mathrm{fe}}\right\}$$

(4) $$f_{c}b_{f}^{\prime }x=f_{y}A_{s}-f_{y}^{\prime }A_{s}^{\prime }+E_{f}\varepsilon_{\mathrm{fe}}A_{f}$$

(5) $$x=\frac{0.8\varepsilon_{\mathrm{cu}}}{\varepsilon_{\mathrm{cu}}+\varepsilon_{\mathrm{fe}}}h$$

where *f_f_* is the tensile strength of the CFRP laminate; *E_f_* is the Young’s modulus of the CFRP laminate; *ε*_fe_ is the effective tensile strain of the CFRP laminate when the concrete strain at the compressive edge reaches the ultimate compressive strain; *ε*_cu_ is the ultimate compressive strain of the concrete, which is taken as 0.0033; and *h* is the height of the RC beam.

For the prestressed CFRP plate, its tensile stress *σ_f_* can be determined using the following equation:

(6) $$\sigma_{f}\;=\min\left\{f_{f},E_{f}\left(\varepsilon_{\mathrm{fe}}+\varepsilon_{\mathrm{fp}0}\right)\right\}$$

where *ε*_fe_ is calculated using Equations (3)–(5) and *ε*_fp0_ is the tensile strain of the prestressed CFRP plate when the stress of the concrete at the bottom edge is zero.

After obtaining the ultimate bending moment, the ultimate load can be calculated based on the following equation:

(7) $$P_{u}=\frac{2M_{u}}{a}$$

where *P_u_* is the ultimate load and *a* is the length of the shear span, i.e., 2300 mm in this study.

The calculated and tested ultimate loads are compared in Table 6, where *δ* represents the relative error between the predicted and tested results. It can be seen that the calculated results agree well with the tested results for the nonprestressed and prestressed CFRP strengthened specimens with concrete crushing failure. However, for specimens with end-debonding failure, the calculated results significantly overestimated the ultimate capacity of the specimens. This further demonstrates that an effective anchorage is critical for the EB–CFRP strengthening technique and premature end-debonding failure should be avoided in practical applications.

## 5. Conclusions

An experimental study was conducted to compare the flexural behavior of RC beams strengthened with nonprestressed and prestressed CFRP materials using different anchorages. The influences of the anchorage method, CFRP type, prestress and prestressing system on the flexural strengthening efficacy were investigated. Based on the limited tests, the following conclusions can be drawn:

(1) Two failure modes were observed for the specimens attributed to the different anchorage methods used. The CFRP end-debonding failure occurred in the specimens anchored by CFRP U-wraps or steel plates. In contrast, the compressive concrete crushing failure occurred in the specimens anchored by wedge-clamp anchors or adhesive-friction anchors.
(2) The flexural strengthening efficacy of the nonprestressed CFRP plate was significantly affected by the anchorage method. The ultimate load and the deformation capacity were remarkably increased while the cracking and yielding loads were limitedly increased when the anchorage method was enhanced from the CFRP U-wraps to wedge-clamp anchors. The strengthening efficacies of the CFRP plate and CFRP sheet were rather close under the same strengthening amount.
(3) Two types of prestressing systems were proved to be effective for flexural strengthening of RC beams using prestressed CFRP plate. The prestress considerably improved the flexural behavior of RC beams. The yielding and ultimate loads were increased by more than 40% and 45%, respectively, using the prestressed CFRP plate. However, the deformation capacity was decreased compared with nonprestressed CFRP strengthening under the same effective anchors.
(4) The CFRP utilization was considerably affected by the anchorage method and prestress. The adequate anchorage of the nonprestressed CFRP could increase the utilization at failure from less than 30% to 59% but had little effect on the utilization at the yielding load, which was only about 16%. However, the CFRP utilization at the yielding load and the failure state could be increased to more than 45% and 70%, respectively, by applying the prestress.

In the practical applications, a reliable anchorage method should be utilized. When the deflection and crack width need to be reduced, the prestressed CFRP strengthening technique is recommended. In addition, because this study only statically tested a limited large-scale RC beams, a systemic numerical studies should be conducted, and fatigue tests need to be carried out to investigate the fatigue behavior of RC beams strengthened with prestressed CFRP plates in the future.

## Figures and Tables

**Table 1:** Main mechanical properties of materials.

Materials	Yielding Stress (MPa)	Ultimate Strength (MPa)	Young’s Modulus (GPa)	Elongation (%)
Steel rebar (*d* = 22 mm)	447	614	202	19.7
Steel rebar (*d* = 25 mm)	452	625	202	26.0
CFRP plate	/	2625	164	1.70
CFRP sheet	/	3870	235	1.73
Adhesive for CFRP plate	/	49.2	4.5	1.64
Adhesive for CFRP sheet	/	60.1	2.9	3.40

Note: *d* is the nominal diameter of the steel rebar.

**Table 2:** Details of the specimens.

Specimens ID	Material Type	Cross-Sectional Size of the CFRP		Designed CFRP Tensioning Strain (με)	Anchorage Method
		Width (mm)	Thickness (mm)		
BU	/	/	/	/	/
BS-1	CFRP plate	100	1.4	/	CFRP U-wraps at two ends
BS-2	CFRP plate	100	1.4	/	Steel plate at two ends and CFRP U-wraps at an intermediate part
BS-3	CFRP plate	100	1.4	/	Wedge-clamp anchors at two ends
BS-4	CFRP sheet	200	0.167×3	/	CFRP U-wraps at two ends and intermediate part
BP-1	CFRP plate	100	1.4	4500	Adhesive-friction anchors at two ends
BP-2	CFRP plate	100	1.4	4500	Wedge-clamp anchors at two ends

**Table 3:** Main test results of the specimens.

Scheme	*P_cr_* (kN)	*α_cr_*	*P_y_* (kN)	*α_y_*	*P_u_* (kN)	*α_u_*	Failure Mode
BU	16	/	193.3	/	221.3	/	Concrete crushing
BS-1	24	1.50	208.6	1.08	220.5	1.00	End-debonding
BS-2	26	1.63	222.6	1.15	246.9	1.12	End-debonding
BS-3	28	1.75	219.6	1.14	305.0	1.38	Concrete crushing
BS-4	24	1.50	221.6	1.15	246.7	1.11	End-debonding
BP-1	42	2.63	265.2	1.37	320.9	1.45	Concrete crushing
BP-2	44	2.75	269.9	1.40	323.1	1.46	Concrete crushing

**Table 4:** Comparisons of the ductility index.

Specimen ID	Δ*_y_* (mm)	Δ*_u_* (mm)	Δ*_u_*/Δ*_y_*
BU	28.5	158	5.54
BS-1	27.5	37.4	1.36
BS-2	27.8	53.0	1.91
BS-3	27.5	157.5	5.73
BS-4	27.7	47.9	1.73
BP-1	31.9	117.0	3.67
BP-2	31.3	113.8	3.64

**Table 5:** Comparisons of CFRP strains at typical loading stages.

Specimen ID	*ε_f,y_* (με)	*ε_f,y_*/*ε_f,u_* (%)	*ε_f,_*_max_ (με)	*ε_f,_*_max_/*ε_f,u_* (%)
BS-1	2707	15.9	4623	27.2
BS-2	2801	16.5	5044	29.7
BS-3	2951	17.4	10032	59.0
BS-4	2477	14.3	4545	26.3
BP-1	7769	45.7	12363	72.7
BP-2	7802	45.9	12497	73.5

**Table 6:** Comparisons of predicted and tested ultimate loads.

Specimen ID	Experimental Results (kN)	Calculated Results (kN)	*δ* (%)
BS-1	220.5	334.0	51.5
BS-2	246.9	334.0	35.3
BS-3	305.0	334.0	9.5
BS-4	246.7	335.8	36.1
BP-1	320.9	356.2	11.0
BP-2	323.1	356.2	10.2

## Data Availability

All data, models, and code generated or used during the study appear in the article.
